# Comparison of the modified S–K method and ICP-MS for urinary iodine measurement: Consistency, accuracy, and vitamin C interference

**DOI:** 10.1097/MD.0000000000045221

**Published:** 2025-11-07

**Authors:** Xiufang Shi, Xiaoqing Zhang, Xuerong Liu, Jiafu Ao

**Affiliations:** a Laboratory Center, Bozhou People’s Hospital, Bozhou City, Anhui Province, China.

**Keywords:** Bland–Altman analysis, ICP-MS, modified S–K method, urinary iodine concentration, vitamin C interference

## Abstract

Iodine status measurement is significant for public health, particularly in pregnant women and individuals with thyroid disease. The current study was to identify the concordance between the modified Sandell–Kolthoff (S–K) assay and inductively coupled plasma mass spectrometry (ICP-MS) assay in measuring urinary iodine concentration, and to evaluate the interference of vitamin C (Vc) level on the measurement accuracy. One hundred fifty-five urine samples were analyzed using both the modified S–K method and ICP-MS with 3 replicate measurements in this prospective study. The samples were categorized into the low Vc level group (qualitative finding of - or ±), the medium Vc level group (qualitative finding of + to 2+), and the high Vc level group (qualitative finding of 3+). Correlation analysis was performed to study the influence of various Vc levels on measuring consistency between the 2 techniques. The modified S–K method showed good overall concordance with ICP-MS (*R* = 0.458, *P* < .01), but the mean urinary iodine concentration measured by the modified S–K method was 38.189 μg/L higher than that of ICP-MS (*P* < .05). Correlation analysis in the different Vc levels showed that with the increasing concentration of Vc, the correlation decreased (*R* = 0.677, 0.655, and 0.494 for low, medium, and high Vc level groups, respectively). Bland–Altman analysis revealed good agreement, but high Vc level concentrations caused positive bias in the modified S–K method. The S–K method altered, though susceptible to interference, particularly from high Vc level, continues to possess superior correlation with ICP-MS.

## 1. Introduction

Iodine is a trace element critical for thyroid hormone formation, which regulates growth, development, and metabolism. Both iodine deficiency and excess can cause thyroid disorders, appearing as goiter, hypothyroidism, and impaired intellectual function. Iodine deficiency is still a significant public health issue, with accurate measurement of iodine nutrition status required for surveillance and successful intervention. Among the available biomarkers, urinary iodine concentration (UIC) is far and away the most widely used with a high-degree-of-trust marker of previous iodine nutrition in clinical studies and also in population surveys.^[1–3]^

UIC has traditionally been assessed with a gold-standard method using the Sandell–Kolthoff (S–K) procedure in virtue of economy, convenience, and accessability.^[4,5]^ It does have many disadvantages to this method, however. It is manual, involves several steps, and contains risky chemicals like arsenic and cerium, which are dangerous to the health of laboratory workers. Moreover, it is prone to interference from reducing agents like vitamin C (Vc), which makes its accuracy debatable. These limitations prevent it from being used on a large scale in studies and clinics, where safety and speed are paramount.^[5]^

Inductively coupled plasma mass spectrometry (ICP-MS) is a highly accurate substitute for UIC measurement and enjoys several advantages over the S–K method. ICP-MS is more sensitive, has a lower detection limit, and less matrix dependence on complex sample matrices.^[4,5]^ There are fewer manipulations, fewer human errors, and more reproducibility. Due to greater sensitivity, ICP-MS is the current gold-standard trace element analysis, including iodine.^[6]^ Huang et al^[7]^ validated ICP-MS for the determination of UIC, with exceptional precision and reproducibility, and it can be a useful alternative to the S–K method in large population-based studies. Ittermann et al^[3]^ also had high variation between laboratories in measuring UIC, and for achieving uniformity in population-based research, there is an urgent requirement for standard iodine assessment procedures. However, the cost being high and advanced equipment required for ICP-MS limits its use in poverty-stricken populations.^[8,9]^

Although both procedures are in general use, their performance has never been systematically compared, especially for large-scale population-based studies. This is crucial because differences in methodology could impact their application to clinical and public health practice. Additionally, concerns regarding the safety and environmental implications of hazardous chemicals in the S–K procedure.^[9–12]^ To combat these problems, our hospital has devised an S–K method modified to replace arsenious acid with antimonous acid, reducing chemical hazards without compromising the efficacy of the method. There is limited data on comparing the S–K method after modification with ICP-MS, particularly concerning reliability and precision.^[8,13]^

This research aims to overcome this limitation by determining the accuracy and reliability of the S–K method altered in relation to ICP-MS. The findings will provide valuable information on the effectiveness of each technique such that the most efficient, safe, and reliable technique can be selected for the determination of UIC. This research, in the long term, will result in enhanced iodine nutritional assessment allowing for more effective public health and clinical interventions against iodine deficiency worldwide.

## 2. Materials and methods

### 2.1. Study participants

This prospective study was approved by the Ethical Committee of Bozhou People’s Hospital (2024-015) and adhered to the Declaration of Helsinki. All the participants have signed written informed consent for this study.

A total of 155 volunteers were recruited from Bozhou People’s Hospital between October 2024 and February 2025. The subjects aged 18 to 65 years were allocated equally between male and female. The inclusion criteria were that the subjects should be in good general health, without any history of thyroid disease or conditions affecting iodine metabolism. The exclusion criteria were recent use of iodine-containing medication or supplements, pregnancy, lactation, and severe hepatic or renal dysfunction.

Basic demographic information (name, sex, and age) and sample collection times were recorded for all participants.

### 2.2. Samples collection

Midstream urine samples were collected in sterile tubes to avoid cross-contamination during collection and processing. The supernatant was collected from the urine samples and then centrifuged at a 3500 rpm for 10 minutes within 2 hours after collection of urine. Then the samples were stored into a 1.5 mL EP tube and was stored in a light-proof box at −70℃ until the time of analysis to ensure sample integrity. The supernatant was stored with no more than 30 days. When the supernatant samples were detected, they should be placed at room temperature to melt for 30 minutes. The same skilled laboratory technicians strictly followed the standardized operation procedures to carry out the test. All analyses were conducted by experienced laboratory personnel utilizing standardized protocols.

The instrument used was the EPNK-600 fully automatic urine biochemical analyzer, and the reagent used was the Ipnokang urine Iodine (I) detection kit. The standard product used the multi-point calibration of Ipnokang urine Iodine standard conduct, and the quality control product used Ipnokang urine iodine quality control product. After calibration, quality control products were tested, and quality control was conducted on standard substances and samples after calibration.

### 2.3. Methodology

UIC were determined by 2 standardized protocols: modified S–K and ICP-MS protocols.

The modified S–K method, which is antimonous acid-catalyzed spectrophotometry, was performed on the EPNK-600 automated urine biochemical analyzer. The instrument used was the EPNK-600 fully automatic urine biochemical analyzer, the reagent was the Ipnokang urine Iodine (I) detection kit, the standard was the Ipnokang urine iodine standard conduct multi-point calibration, and the quality control product was the Ipnokang urine iodine quality control product. After calibration, quality control products are tested, and quality control is conducted on standard substances and samples after calibration.

The ICP-MS method was performed on the Inspector SQ60 trace element analyzer (Reliable Med, Hangzhou, China; Website: http://www.reliablemed.cn/). The iodine element detection material package (including calibrators, internal standard solutions, and quality control products), iodine element sample dilution solution, tuning solution, cleaning solution, 5 mL centrifuge tubes, pipettes with appropriate volume range and corresponding suction tips, and vortex mixers were all purchased from Reliable Med. The experimental steps are as follows: the components in the consumable package were taken out from 2 to 8℃ storage and were placed at room temperature for 10 minutes; a multi-tube vortex mixer was used to shake the sample for 1 minute at 2500 rpm. A total of 400 μL of the internal standard was added to the newly opened sample diluent, and a multi-tube vortex mixer was used to shake the mixed liquids at 2500 rpm for 5 minutes for later use. A 2.8 mL of the above sample mixment was added to a 5 mL centrifuge tube, and was mixed with a 200 μL of the samples (including the blank sample, calibrators 1–6, urine sample, and quality control product). All the mixed samples were shaken at 2500 rpm for 5 minutes before testing on the machine. According to the contents in the “User Manual of Trace Element Analyzer” of Inspector SQ60, the tuning and adjustments of the parameters were performed on Inspector SQ60 to meet the requirements of the experiments. The blank sample, calibrators 1 to 6, quality control product, and the waiting-test samples were tested simultaneously, and the calibration curves were drawn automatically. At the same time, the concentration of the element to be measured in the sample is automatically calculated through the software. When calibrating the instrument with calibrators and internal standards, the system automatically plots the calibration curve. When the calibration curve does not meet the requirements (the *r*^2^ of the calibration curve should be ≥ 0.990), the instrument status needs to be investigated and recalibrated. After calibration is passed, quality control products are tested. Quality control then conducts standard substance and sample testing.

According to ICP-MS and the modified S–K and protocols, the urinary iodine concentration levels of the collected samples, and the included national primary standard substances for urinary iodine (GBW09109n and GBW09110i) were detected respectively using the Inspector SQ6 trace element determination analyzer, and the EPNKI-600 fully automatic urine biochemical analyzer respectively. The measurement values of each time were recorded. The standard substances (GBW09109n and GBW09110i) were measured at least 6 times, and the collected samples should be retested at least for 3 times for increased reliability and reproducibility of reading.

The samples were grouped based on the urinary Vc levels (urinary dry chemistry detection) into 3 groups: the low Vc level group (negative or ±), the medium Vc level group (+ to 2+), and the high Vc level group (3+). The data among the 3 groups were compared.

### 2.4. Precision testing

Precision testing was conducted in accordance with the standards of “CLSI EP15-A3-2014, YY1789.1-2021 Evaluation Method for Performance of In Vitro Diagnostic Testing Systems – Part 1: Precision.” Tests are conducted in the same laboratory by the same operator on the same instrument. Two concentrations of urinary iodine quality control products, high and low, were used respectively, and 15 tests were conducted in different batches. Take 1/2 TEA as the evaluation criterion. The improved S–K method uses EPNK quality control products (high and low level quality control products with batch number 20231227), while the ICP-MS method uses RLP quality control products from Ruilepu (high and low level quality control products with batch number 324702).

### 2.5. Statistical analysis

Data were analyzed using SPSS 27.0 (IBM Corp., Armonk). The Kolmogorov–Smirnov (K–S) test was employed to test normality of the data. Normally distributed continuous data were expressed as mean ± standard deviation, and skewed data were reported as median (P25, P75). Groups were compared using independent t-tests or nonparametric rank-sum tests, as appropriate. Spearman correlation analysis was conducted to identify relationships between variables. ICP-MS and the S–K method were compared with Bland–Altman plots, which calculated the 95% limits of agreement. The plots give a visual comparison of the difference between the methods and identify any systematic bias or variation. Statistical significance was taken as a *P*-value of <.05, such that differences between methods were unlikely to occur by chance. This systematic review gave a complete overview of the validity and reproducibility of both methods employed to quantify urinary iodine.

## 3. Results

### 3.1. Accuracy comparison of UIC measurements using the modified S–K method and ICP-MS

Accuracy verification checks conformity between instrument measurements and true values assigned. The research in this study followed the YY/T 1789.2-2021 standard (In Vitro Diagnostic Test Systems-Performance Evaluation Methods: Part 2: Accuracy)^[14]^ with certified reference materials traceable and interchangeable. Both the S–K method and ICP-MS were used to measure concentrations of iodine in lyophilized human urine reference materials (GBW09109n and GBW09110i). Each reference material was measured 6 times within 72 hours to determine the accuracy of measurements.

Table 1 presents UIC measurements for the 2 certified reference materials using both methods. The values were compared to standard reference values (116.000 for GBW09109n and 226.000 for GBW09110i). The ICP-MS method was less biased and variable, with values nearer to the standard values. The modified S–K method overestimated GBW09109n slightly (mean = 124.284) and underestimated GBW09110i slightly (mean = 220.369). For GBW09109n, the modified S–K method’s mean was 124.284, with 7.14% bias relative to ICP-MS (mean = 115.717). For GBW091110i, the modified S–K process meant 220.369, and 2.49% bias relative to ICP-MS (meaning = 223.017). The results indicate the relative stability of procedures with minor bias for the modified S–K process specifically for GBW09109n. This urine indicates the accuracy and accuracy of the revised S–K process for iodine assessment.

**Table 1:** Accuracy comparison of UIC measurements using the modified S–K method and ICP-MS.

Method	Reference material	Detection 1	Detection 2	Detection 3	Detection 4	Detection 5	Detection 6	Mean	Standard value	Difference	Bias (%)
Modified S–K	GBW09109n	125.150	126.998	127.011	120.865	123.349	122.331	124.284	116.000	8.284	7.14%
Modified S–K	GBW09110i	216.417	217.806	222.345	220.182	221.320	224.145	220.369	226.000	5.631	2.49%
ICP-MS	GBW09109n	113.817	115.733	116.754	115.231	115.484	117.284	115.717	116.000	0.283	-0.24%
ICP-MS	GBW09110i	220.684	221.846	217.850	225.213	228.151	224.361	223.017	226.000	2.983	-1.32%

ICP-MS = inductively coupled plasma mass spectrometry, S–K = Sandell–Kolthoff, UIC = urinary iodine concentrations.

However, ICP-MS is also more expensive and requires specialized hardware, thus is limited in some configurations. Despite its limitations, the S–K method modification remains an appropriate alternative to routine urinary iodine testing for resource-poor environments.

### 3.2. Results of precision analysis

Precision testing results were shown in Table 2.

**Table 2:** Precision tests results.

	Modified S–K (Batch number: 2023122)		ICP-MS (Batch number: 324702)	
	L1	L2	L	H
Detection 1	103.20	230.00	393.9084	499.5381
Detection 2	119.70	245.10	400.8911	512.3102
Detection 3	104.70	238.90	392.5056	518.5645
Detection 4	122.00	203.70	439.9145	561.2356
Detection 5	114.30	204.20	438.8676	559.5116
Detection 6	98.70	195.30	438.2567	558.1603
Detection 7	114.40	192.00	399.9639	557.6584
Detection 8	98.70	195.30	405.8284	557.188
Detection 9	114.40	192.00	411.0258	556.8946
Detection 10	104.20	185.10	377.0324	528.0653
Detection 11	108.40	177.20	404.8942	533.1999
Detection 12	107.60	186.90	426.8869	552.2489
Detection 13	103.60	186.20	417.9015	487.6801
Detection 14	107.50	184.20	374.4427	511.8777
Detection 15	102.90	187.40	390.8112	503.3221
Mean	108.29	200.23	407.54	533.16
Standard deviation	6.93	20.27	20.49	25.07
Coefficient of variation	6.40%	10.12%	5.03	4.70
Whether to accept	Accept	Accept	Accept	Accept

ICP-MS = inductively coupled plasma mass spectrometry, S–K = Sandell–Kolthoff.

### 3.3. Results of normality test analysis

Normality of UIC values was tested by K–S test and Shapiro–Wilk (S–W) test (Table 3). For the S–K method altered, the K–S test (*D* = 0.081, *P* = .015) and S–W test (W = 0.958, *P* < .01) showed considerable deviation from normality. The ICP-MS method showed even greater deviation (*D* = 0.195, *P* < .01, *W* = 0.628, *P* < .01) with extreme skewness (4.352) and kurtosis (27.409). These results confirm that both sets of data are not normally distributed and, therefore, the use of nonparametric statistical methods in analysis is warranted.

**Table 3:** Normality test results for UIC measurements using the modified S–K method and ICP-MS.

Method	Sample size (n)	Mean	Standard deviation	Skewness	Kurtosis	Kolmogorov–Smirnov test	Shapiro–Wilk test
Modified S–K	155	191.319	79.177	0.673	0.542	*D* = 0.081, *P* = .015*	*W* = 0.958, *P* < .01*
ICP-MS	155	153.130	111.075	4.352	27.409	*D* = 0.195, *P* < .01*	*W* = 0.628, *P* < .01*

ICP-MS = inductively coupled plasma mass spectrometry, S–K = Sandell–Kolthoff, UIC = urinary iodine concentrations.

* *P* < .05.

### 3.4. Difference analysis test results

Wilcoxon paired sample test indicated that there was a statistically significant difference between the modified S–K technique and ICP-MS (Table 4). The median of the modified S–K technique (183.300) was statistically higher than the median of the ICP-MS technique (126.919), that is, the higher values are obtained with the modified S–K technique. Statistical significance is confirmed by the *Z*-value (6.085) and the *P*-value (<.001), which is <.05, thus supporting the conclusion that the observed difference is statistically significant.

**Table 4:** Wilcoxon paired sample analysis results.

Name	Paired median M (P25, P75)	Median M difference (Pair 1–Pair 2)	*Z*-value	*P*-value
Modified S–K	183.300 (124.9, 245.4)	56.381	6.085	<.001*
ICP-MS	126.919 (98.9, 171.2)			

ICP-MS = inductively coupled plasma mass spectrometry, S–K = Sandell–Kolthoff.

* *P* < .05.

### 3.5. Consistency analysis test results

**Figure 1.**
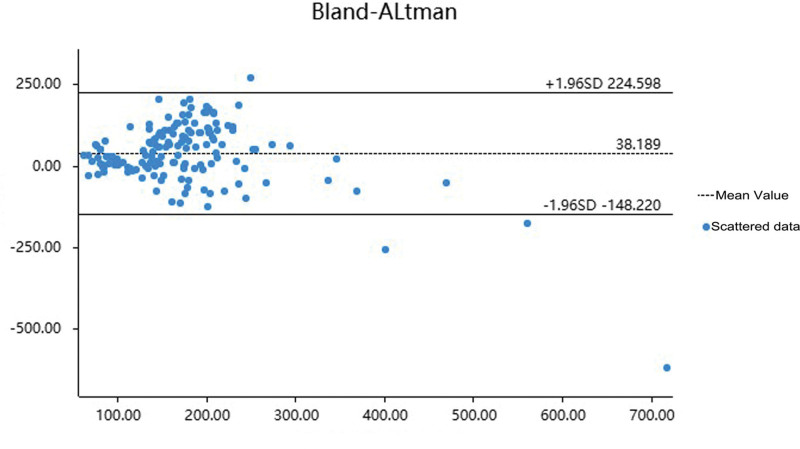
Comparison of mean values between ICP-MS and modified S–K Method. ICP-MS = inductively coupled plasma mass spectrometry, S–K = Sandell–Kolthoff.

Consistency between the techniques was tested via Bland–Altman analysis. The difference between the methods averaged 38.189 and had a 95% confidence interval of between 23.098 and 53.281, which presents a significant difference. The variability of the differences was 95.108, which shows variations in the data, though the majority of measurements are near to the mean difference. The 95% confidence interval of differences ranged between −48.220 and 224.598, indicating most data points within the acceptable limit (±1.96 standard deviation), but outliers existed. *t*-value of 4.999 and *P*-value of .000 confirmed that the mean difference was statistically significant. Coefficient of repeatability was 200.316, indicating good repeatability and consistency in most cases. In general, the analysis proved to be consistent across the methods but further study of the outliers might provide more information on the discrepancies (Table 5 and Fig. 1).

**Table 5:** Bland–Altman descriptive statistics.

Items	Values
Effective sample size	155
Mean (Method 1)	191.319
Mean (Method 2)	153.130
Mean difference	38.189
Standard deviation (difference)	95.108
95% CI (mean difference)	23.098–53.281
95% CI (difference)	-48.220 to 224.598
*t*-value (H0: mean difference = 0)	4.999
*P*-value (H0: mean difference = 0)	<.001*
CR value (coefficient of repeatability)	200.31

CI = confidence interval, CR = coefficient of repeatability.

* *P* < .05.

### 3.6. Correlation analysis results

#### 3.6.1. Correlation analysis between the 2 detection methods

Spearman correlation between the 2 methods’ results indicated moderate positive correlation (*R* = 0.458), with statistically significant *P*-value (*P* < .01), indicating moderate positive linear correlation between the 2 methods (Table 6).

**Table 6:** Correlation between the modified S–K and ICP-MS methods across different urinary Vc level groups.

Method 1	Method 2	Correlation coefficient (*r*)	*P*-value	Sample size
Modified S–K	ICP-MS	0.458	<.01*	155
Modified S–K†	ICP-MS	0.494	<.001*	43
Modified S–K‡	ICP-MS	0.655	<.001*	51
Modified S–K§	ICP-MS	0.677	<.001*	61

ICP-MS = inductively coupled plasma mass spectrometry, S–K = Sandell–Kolthoff, Vc = vitamin C.

† High Vc level group.

‡ Medium Vc level group.

§ Low Vc level group.

* *P* < .05.

#### 3.6.2. Correlation among different Vc levels

The subjects were divided into 3 groups based on urinary Vc levels: low Vc level, mediumVc level, and high Vc level. Spearman rank correlation analysis of ICP-MS and modified S–K method results showed correlation coefficients of 0.494 in the high Vc level group, 0.655 in the medium Vc level group, and 0.677 in the low Vc level group (Table 6). All correlations were highly significant (*P* < .001) and increased in strength as Vc levels decreased. The lowest Vc level group presented the highest correlation, followed by the medium and high Vc level groups. From the results, it can be seen that more elevated urinary Vc level may present a slightly reduced agreement between the methods.

## 4. Discussion

Nutritional iodine is of paramount significance, especially among vulnerable groups such as pregnant women and patients with thyroid dysfunction. One of the key issues is how to introduce targeted interventions and give proper iodine supplementation to different groups of populations.^[9,10,15]^ Insufficient iodine and thyroid disorders in pregnancy are key determinants of adverse maternal and fetal outcomes and therefore require sufficient iodine supplementation and thyroid surveillance.^[16]^

No consensus exists regarding the best methods and criteria for the assessment of iodine status. UIC remains the most widely applied metabolic indicator of iodine nutrition and is universally accepted as the indicator of choice for the evaluation of iodine nutrition status.^[17]^ The S–K reaction has been widely applied to iodine monitoring but requires complex sample preparation and employs hazardous reagents like arsenic and cerium, which are biosafety hazards. Further enhancements have been aimed at improving accuracy, safety, and efficiency. Additional modifications of S–K technology, such as replacement of dangerous chemicals, are also ready to adapt to laboratory safety without renouncing cost-effectiveness. The ICP-MS was also adapted to become more accurate and the sample complexity has a low detection limit for the amount of trace of iodine in complexity. Grimm et al^[18]^ described a micro-photometric simplified method for urinary iodine determination, an economical and efficient option to ICP-MS for routine clinical use in iodine screening. ICP-MS has sensitivity, good anti-interference properties, and good reproducibility, and as such, increasingly is encouraged in large population investigations.^[19]^ However, owing to high technology demands and lack of quick reimbursement by health insurers, extensive clinic application remains confined.

This research applied a modified S–K technique, substituting antimonous acid for arsenious acid to minimize chemical risk without loss of measurement precision. Our findings illustrated that ICP-MS had negligible bias at both concentration levels, which reflected its high precision. UIC values from both techniques were not normally distributed, and the difference between them was significant (*P* < .05). The mean UIC value derived with the S–K method modified as above was 38.189 μg/L higher than for ICP-MS, with the difference proving statistically significant (*P* < .05). Bland–Altman plots showed there was good correlation between the methods.

Li et al^[8]^ compared ICP-MS with S–K method in a comparative study and concluded that ICP-MS gave a high level of UIC to a high level, especially at a high level of iodine. His study reported a difference of 6.12 μg/L between 2 methods, mainly on UIC values > 300 μg/L, and recommended the application of ICP-MS for accurate monitoring in iodine nutrition studies. The difference was even greater at > 600 μg/L, showing that ICP-MS is more accurate, especially in groups with increased iodine intake. While our study showed excellent general agreement between ICP-MS and the modified S–K method, the mean difference was greater than previously reported study.^[8]^ Such disparity can occur since interference from urinary substances in the modified S–K method is higher, something which highlights its drawbacks. Oblak et al^[20,21]^ validated the adapted S–K method for measuring urinary iodine in microplates, with the result being comparable to ICP-MS and suitable for routine laboratory determination of iodine. Meticulous reagent and sample preparation is important, as substances like phosphates, fluorides, and oxidizing substances affect the results of the S–K test. Drugs and baselines may also affect measurements.^[20,21]^ This study used ammonium persulfate for digestion, which may produce differences in S–K method results.

In the current work, 155 samples found a Spearman correlation between the modified S–K technology and ICP-MS, which represents a moderate positive linear correlation (*R* = 0.458) between 2 techniques. Nevertheless, the strength of the correlation was relatively low. As previously stated, the modified S–K approach is prone to interference, especially from inorganic compounds, though these are not common in urine.^[20,21]^ The principal organic interfering compound for UIC is Vc.^[22]^ High levels of Vc enhance cerium oxidation in the S–K reaction, leading to positive interference and falsely elevated urinary iodine values.^[23]^ This situation can also explain why 2 methods are usually comparable with good consistency between 2 groups but with significant differences in mean values in our study. As high Vc level could spuriously increase iodine values, dividing samples into low, medium, and high Vc level groups allows us to assess the impact of varying Vc content on measurement reliability, identify interferences at thresholds, and increase method robustness. Our results indicated that as the Vc concentration increased, the correlation between both methods declined, suggesting that the high levels of Vc interfere with modified S–K method. Furthermore, Ornella Joseph et al^[22]^ also identified urobilin and cysteine as the main positive interfering UIC detection in the S–K method. The current study only collected the data of Vc interference on UIC and did not analyze urobilin and cysteine, which might have caused the significant differences in the mean values between the 2 groups. The interference urobilin and cysteine on UIC awaits further study.

Previous evidence shows that there are significant differences in group iodine nutrition among countries and regions. These differences are caused by factors such as the coverage rate of iodized salt, relevant legislation and implementation of iodine nutrition, as well as the dietary structure and water source background of residents.^[24]^ Moreover, the iodine nutritional thresholds required by different groups of people, such as school-age children,^[25]^ women of childbearing age/pregnant women,^[10,15,26]^ and adults,^[27]^ also vary. Therefore, the distribution of urinary iodine among people in different regions is not consistent. Meanwhile, the nutritional status of Vc also varies with regional factors, behavioral factors and socioeconomic levels.^[28]^ Low-income people^[29]^ and smokers^[30]^ have a higher risk of Vc deficiency. Therefore, the “positive proportion of Vc” and its intensity in samples from different regions may vary. All of the above factors may affect the potential interference degree of Vc in the improved S–K and the estimation results of inter-method bias.

This study has a few limitations. First, the study analyzes 155 urine samples from a single population, which may limit the generalizability of the findings to broader populations with diverse nutritional, geographic, or socioeconomic backgrounds. Second, this study had potential interference from urinary constituents, especially from excessive Vc, and Vc varies in different regions and different populations. Clinical practice should consider the Vc interference. Third, the lack of longitudinal data limits consideration of reproducibility over the longer term, and application of a single digestion methodology may limit accuracy. Fourth, UIC is influenced by urine dilution, which can vary widely between individuals. The absence of creatinine-adjusted UIC values or consideration of urine specific gravity may impact the accuracy and comparability of the results. Therefore, the results of this study are first recommended to be applied to the regions or environments with iodine and Vc conditions similar to that in this study and the similar population. For populations with other nutritional, socioeconomic lineages and different Vc interference, multi-center validation should be conducted (prioritizing those with different iodized salt coverage levels and different smoking/income structures).^[24–30]^ It is also recommended to report consistency indicators for different populations simultaneously. Moreover, subsequent research needs to focus on exploring other digestion techniques, measuring long-term stability, and considering adjustments of creatinine-adjusted UIC values or consideration of urine specific gravity.

## 5. Conclusion

While the S–K method with modifications is subject to interference from numerous factors and therefore leads to some inconsistency in urinary iodine determination, it is still highly consistent in general with ICP-MS. Notably, if high Vc level samples are omitted, the modified S–K method provides consistent results. The modified S–K method can be utilized as an invaluable tool for clinical screening, uses, and research in iodine nutrition.

## Author contributions

**Conceptualization:** Xiufang Shi, Jiafu Ao.

**Data curation:** Xiaoqing Zhang, Xuerong Liu.

**Formal analysis:** Xiaoqing Zhang, Xuerong Liu.

**Funding acquisition:** Jiafu Ao.

**Resources:** Xiufang Shi, Xuerong Liu.

**Supervision:** Xiufang Shi, Jiafu Ao.

**Visualization:** Xiaoqing Zhang.

**Writing – original draft:** Xiufang Shi, Jiafu Ao.

**Writing – review & editing:** Xiufang Shi, Xiaoqing Zhang, Xuerong Liu, Jiafu Ao.

## References

[1] Mao GM Mo Z Gu SM. Analysis of iodine nutritional status in children aged 8–10 years in Zhejiang Province from 2016 to 2021. Chin J Prev Med. 2024;58:11–7. 10.3760/cma.j.cn112150-20230707-00524 38228544

[2] Zha H Yu L Tang Y Sun L Yuan Q. Effect of iodine nutrition status on thyroid function and pregnancy outcomes. Biol Trace Elem Res. 2023;201:5143–51. 36763262 10.1007/s12011-023-03600-8

[3] Ittermann T Johner S Below H. Interlaboratory variability of urinary iodine measurements. Clin Chem Lab Med. 2018;56:441–7. 28941352 10.1515/cclm-2017-0580

[4] Zhou Q Xue S Zhang L Chen G. Trace elements and the thyroid. Front Endocrinol (Lausanne). 2022;13:904889. 36353227 10.3389/fendo.2022.904889 PMC9637662

[5] Jooste PL Strydom E. Methods for determination of iodine in urine and salt. Best Pract Res Clin Endocrinol Metab. 2010;24:77–88. 20172472 10.1016/j.beem.2009.08.006

[6] Xu SD Xie JA Ding G. Study on the determination of urinary iodine using antimony-cerium catalytic spectrophotometry. Chin J Endemiol. 2024;43:750–4.

[7] Huang CJ Lee LH Cheng CP. Analytical validation of an inductively coupled plasma mass spectrometry method for urinary iodine concentration measurements in Taiwan. J Formos Med Assoc. 2023;122:757–65. 36878768 10.1016/j.jfma.2023.02.010

[8] Li Y Ding S Han C. Concentration-dependent differences in urinary iodine measurements between inductively coupled plasma mass spectrometry and the Sandell–Kolthoff method. Biol Trace Elem Res. 2021;199:2489–95. 33034809 10.1007/s12011-020-02381-8 PMC8213661

[9] Liu P. Precision iodine supplementation and the establishment of the iodine nutrition evaluation system. Chin J Endemiol. 2022;41:609–12.

[10] Amouzegar A Khazan M Hedayati M Azizi F. An assessment of the iodine status and the correlation between iodine nutrition and thyroid function during pregnancy in an iodine sufficient area. Eur J Clin Nutr. 2014;68:397–400. 24398634 10.1038/ejcn.2013.273

[11] Macours P Aubry JC Hauquier B Boeynaems JM Goldman S Moreno-Reyes R. Determination of urinary iodine by inductively coupled plasma mass spectrometry. J Trace Elem Med Biol. 2008;22:162–5. 18565428 10.1016/j.jtemb.2008.02.003

[12] Shelor CP Dasgupta PK. Review of analytical methods for the quantification of iodine in complex matrices. Anal Chim Acta. 2011;702:16–36. 21819856 10.1016/j.aca.2011.05.039

[13] Wilschefski SC Baxter MR. Inductively coupled plasma mass spectrometry: introduction to analytical aspects. Clin Biochem Rev. 2019;40:115–33. 31530963 10.33176/AACB-19-00024 PMC6719745

[14] Beijing Medical Device Testing Institute, Meikang Biotechnology Co., Ltd., and Hissen Meikang Medical Electronics Co., Ltd., . In vitro diagnostic test systems – performance evaluation methods – Part 1: precision. National Medical Products Administration; 2021: 28.

[15] Chen X Wu C Wang Z. Iodine nutrition status and thyroid autoimmunity during pregnancy: a cross-sectional study of 4635 pregnant women. Nutr J. 2022;21:7. 35093086 10.1186/s12937-022-00760-6 PMC8801104

[16] Sun D Codling K Chang S. Eliminating iodine deficiency in China: achievements, challenges and global implications. Nutrients. 2017;9:361. 28379180 10.3390/nu9040361 PMC5409700

[17] Xia J Yu L Yuan Y. Assessment of iodine nutrition in pregnant women during the second and third trimesters and its relationship with hypothyroidism. J Nanjing Med Univ Nat Sci Ed. 2022;42:1415–1420,1450.

[18] Grimm G Lindorfer H Kieweg H. A simple micro-photometric method for urinary iodine determination. Clin Chem Lab Med. 2011;49:1749–51. 21702698 10.1515/CCLM.2011.653

[19] Yu S Cheng Q Han JH. Establishment of clinical methods for detecting iodine in human urine and serum using inductively coupled plasma mass spectrometry. Chin J Lab Med. 2016;39:917–21.

[20] Oblak A Imperl J Kolar M. Introduction of a spectrophotometric method for salivary iodine determination on microplate based on Sandell–Kolthoff reaction. Radiol Oncol. 2024;58:357–65. 39042833 10.2478/raon-2024-0035 PMC11406899

[21] Oblak A Arohonka P Erlund I Kuzmanovska S Zaletel K Gaberšček S. Validation of a spectrophotometric method for urinary iodine determination on microplate based on Sandell–Kolthoff reaction. Lab. Med.. 2022;53:376–80. 35073580 10.1093/labmed/lmab117

[22] Joseph O Eberle M Lieberman M. Metabolites in urine that interfere with the Sandell–Kolthoff assay for urinary iodine. Biol Trace Elem Res. 2024;202:466–72. 37222924 10.1007/s12011-023-03710-3

[23] Ford HC Johnson LA. Ascorbic acid interferes with an automated urinary iodide determination based on the Ceric–Arsenious Acid reaction. Clin Chem. 1991;37:759. 2032332

[24] Hatch-McChesney A Lieberman HR. Iodine and iodine deficiency: a comprehensive review of a re-emerging issue. Nutrients. 2022;14:3474. 36079737 10.3390/nu14173474 PMC9459956

[25] Qian TT Sun R Liu LC. Relationship between dining place, iodine source, and iodine nutrition in school-age children: a cross-sectional study in China. Biomed Environ Sci. 2023;36:10–23. 36650678 10.3967/bes2023.002

[26] Sonowal T Sarmah J Sarma PK Deka M. Iodine status, thyroid disorder and feto-maternal outcome among the tribal pregnant women of Eastern Himalayas. Indian J Endocrinol Metab. 2023;27:66–72. 37215276 10.4103/ijem.ijem_367_22 PMC10198190

[27] Abuduwaili G Huang J Ma Y Sun H. Adult dietary patterns and their association with iodine nutrition levels and thyroid function: a cross-sectional study. Public Health Nutr. 2024;28:e4. 39600224 10.1017/S1368980024002404 PMC11736648

[28] Passarelli S Free CM Shepon A Beal T Batis C Golden CD. Global estimation of dietary micronutrient inadequacies: a modelling analysis. Lancet Glob Health. 2024;12:e1590–9. 39218000 10.1016/S2214-109X(24)00276-6 PMC11426101

[29] Rowe S Carr AC. Global vitamin C status and prevalence of deficiency: a cause for concern? Nutrients. 2020;12:2008. 32640674 10.3390/nu12072008 PMC7400810

[30] Golder JE Bauer JD Barker LA Lemoh CN Gibson SJ Davidson ZE. Prevalence, risk factors, and clinical outcomes of vitamin C deficiency in adult hospitalized patients in high-income countries: a scoping review. Nutr Rev. 2024;82:1605–21. 38219216 10.1093/nutrit/nuad157 PMC11465154

